# Book review: Advanced Headache Therapy by Lawrence Robbins

**DOI:** 10.1186/s10194-015-0538-0

**Published:** 2015-06-10

**Authors:** Duren Michael Ready

**Affiliations:** Baylor Scott & White Central Division, Temple, Texas

**Keywords:** Outpatient headache treatment, Refractory migraine, Migraine comorbidities, Cluster headache, Adolescent migraine

## Abstract

Advanced Headache Therapy (Outpatient Treatment): Review

Springer 2015

ISBN-13 978–3319138985

Advanced Headache Therapy, is Dr. Robbins 4th headache book and it fully covers “standard” outpatient headache treatment. He discusses many non-pharmacological concepts such as resilience, acceptance, and interventions that are beyond the “pill for every ill” mentality. Standard medications are extensively covered. However, the strength of this book is “what to do when nothing works”.

The author analyzes why patients fail usual treatment, and he delves deeply into the psychiatric comorbidities. He analyzes the effect of psychiatric conditions on headache treatment. There are lengthy sections on recognition of the bipolar spectrum, along with the various personality disorders. Anxiety and ADHD are thoroughly covered.

One long and interactive case history involves the complex treatment of a young woman over ten years time. She has bipolar and severe chronic migraine, and the multiple treatment options are debated. This is a remarkable “real life” journey through a young woman’s 20’s, one that we often grapple with in our clinics.

Complete guidelines for treating children and adolescents are presented. There is a fascinating case discussion of homebound adolescents. Another interesting case involves adolescents and “Mild Munchausens by Proxy”.

While refractory chronic migraine is the primary focus, cluster headache is also addressed. There are also interesting and practical cluster cases. Dr. Robbins weighs in on migraine surgery (he is negative on it). He also presents a compelling case for the use of opiates and butalbital containing medication in a subset of otherwise refractory patients. But most importantly (in my mind) he outlines how to determine which patients are likely to benefit from these medications.

The extensive refractory sections include the introduction of a “refractory scale”, definitions for refractory chronic migraine, and many treatment options. These include MAOI’s, daily triptans, Botox, SPG blocks, opioids, and stimulants. Dr. Robbins excels at “thinking outside the box”.

Additional sections include: clinical pearls, the immune system and headache, chaos theory and headache, a comprehensive section on NDPH, and medication overuse headache. Dr. Robbins often takes a contrarian view, and he feels that medication overuse is poorly defined and over-diagnosed.

Advanced Headache Therapy represents principally Dr. Robbins opinion, which is both a strength and a negative. It would have been helpful if there were discussions of inpatient treatments as well, as the focus is all outpatient. A separate section on treating the elderly would have been useful. Dr. Robbins, who has published extensively on refractory headaches, has written a practical “real world” guide to outpatient treatment. It is a welcome addition to my library.

